# Peroxygenase-Catalyzed Allylic Oxidation Unlocks Telescoped Synthesis of (1*S*,3*R*)-3-Hydroxycyclohexanecarbonitrile

**DOI:** 10.1021/acscatal.4c00177

**Published:** 2024-02-13

**Authors:** Christian M. Heckmann, Moritz Bürgler, Caroline E. Paul

**Affiliations:** † Biocatalysis section, Department of Biotechnology, Delft University of Technology, van der Maasweg 9, 2629HZ Delft, The Netherlands; ‡ Bisy GmbH, Wünschendorf 292, 8200 Hofstätten an der Raab, Austria

**Keywords:** biocatalysis, ene reductase, alcohol dehydrogenase, peroxygenase, cascade, retrosynthesis

## Abstract

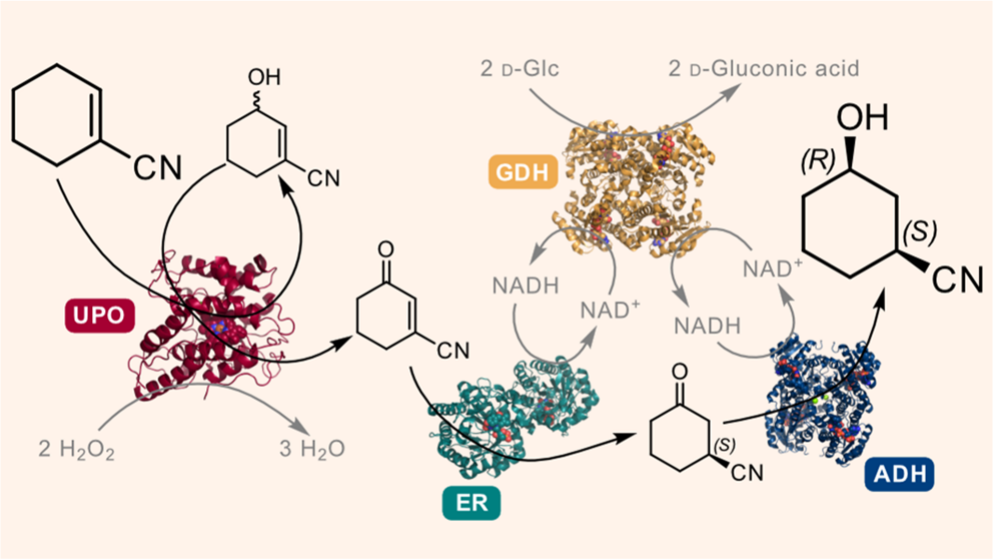

The unmatched chemo-, regio-, and stereoselectivity of enzymes renders them powerful catalysts in the synthesis of chiral active pharmaceutical ingredients (APIs). Inspired by the discovery route toward the LPA_1_-antagonist BMS-986278, access to the API building block (1*S*,3*R*)-3-hydroxycyclohexanecarbonitrile was envisaged using an ene reductase (ER) and alcohol dehydrogenase (ADH) to set both stereocenters. Starting from the commercially available cyclohexene-1-nitrile, a C–H oxyfunctionalization step was required to introduce the ketone functional group, yet several chemical allylic oxidation strategies proved unsuccessful. Enzymatic strategies for allylic oxidation are underdeveloped, with few examples on selected substrates with cytochrome P450s and unspecific peroxygenases (UPOs). In this case, UPOs were found to catalyze the desired allylic oxidation with high chemo- and regioselectivity, at substrate loadings of up to 200 mM, without the addition of organic cosolvents, thus enabling the subsequent ER and ADH steps in a three-step one-pot cascade. UPOs even displayed unreported enantioselective oxyfunctionalization and overoxidation of the substituted cyclohexene. After screening of enzyme panels, the final product was obtained at titers of 85% with 97% *ee* and 99% *de*, with a substrate loading of 50 mM, the ER being the limiting step. This synthetic approach provides the first example of a three-step, one-pot UPO-ER-ADH cascade and highlights the potential for UPOs to catalyze diverse enantioselective allylic hydroxylations and oxidations that are otherwise difficult to achieve.

## Introduction

**Scheme 1:**
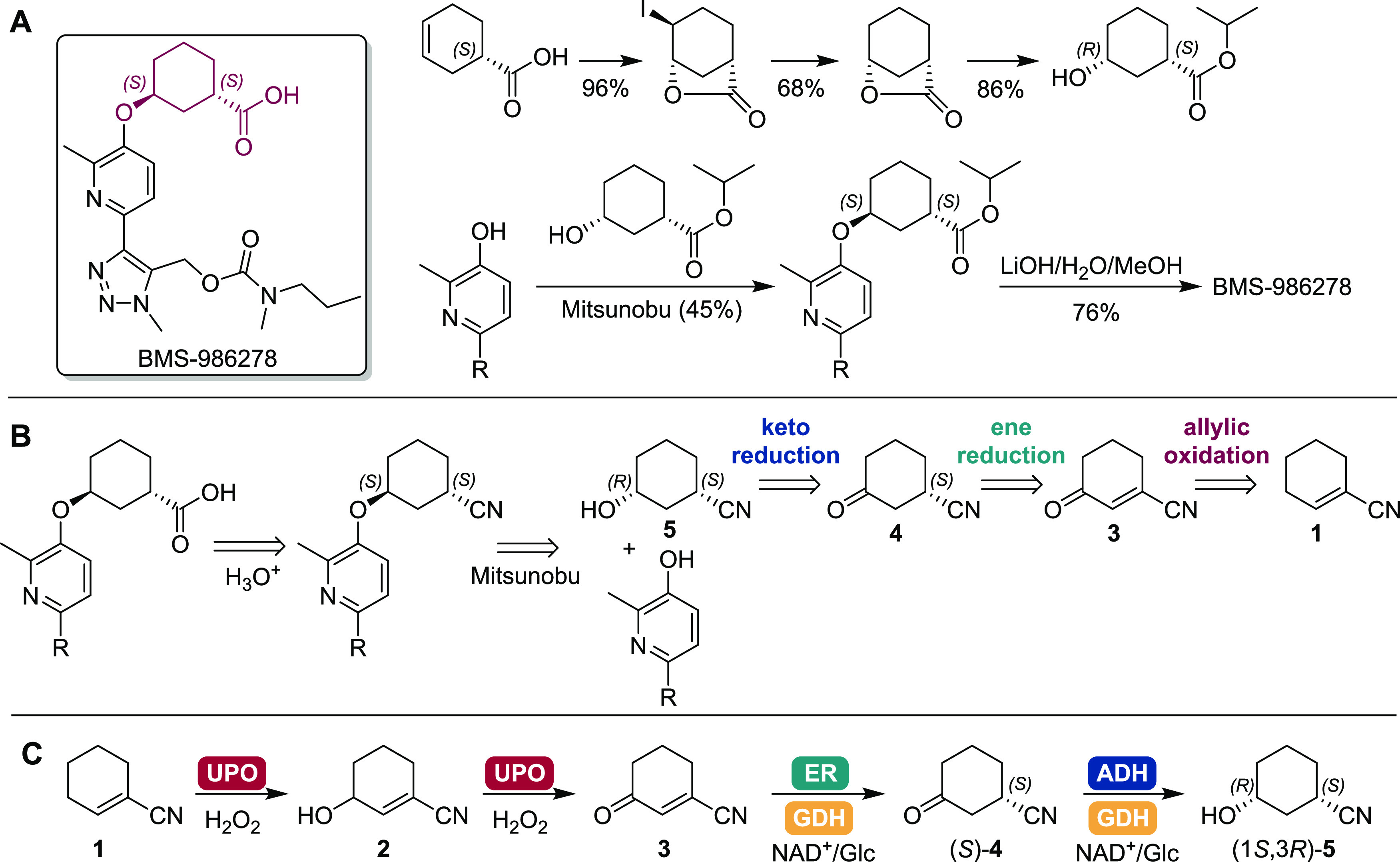
(A) Abbreviated Discovery Route to BMS-986278 and Retrosynthetic Analysis (A) Abbreviated discovery route to BMS-986278. A chiral substituted cyclohexanol is prepared from (*S*)-cyclohex-3-enecarboxylic acid, which is then coupled to the pyridine moiety and deprotected to give BMS-986278. (B) Retrosynthetic Analysis of a Simplified BMS-986278 Analogue. The acid may be obtained by hydrolysis of a nitrile group, which would be compatible with the Mitsunobu reaction. The chiral 3-hydroxycyclohexanecarbonitrile **5** may be prepared from the pro-chiral cyclohex-1-enecarbonitrile **1**. (C) The Proposed Enzymatic Synthesis of (1*S*,3*R*)-**5**, Where UPO = Unspecific Peroxygenase, ER = Ene Reductase, ADH = Alcohol Dehydrogenase, and GDH = Glucose Dehydrogenase.

The field of biocatalysis has revealed the unmatched selectivity of enzymes to access chiral active pharmaceutical ingredients (APIs) with high purity and even as a synthetic shortcut compared with chemical approaches.^1^ In this respect, Bristol Myers Squibb (BMS) recently disclosed the lysophosphatidic acid receptor 1 (LPA_1_) antagonist BMS-986278 (Scheme 1), which is currently in phase II clinical trials for the treatment of idiopathic pulmonary fibrosis.^2^ BMS-986278 contains two stereocenters, which during the discovery synthesis (Scheme 1A) were derived in diastereoselective fashion from the chiral starting material (*S*)-3-cyclohexene carboxylic acid, requiring classical resolution of its racemate, and being inherently wasteful. Thus, an alternative synthesis from a pro-chiral starting material would be preferable, but achieving high stereopreference in chemical reactions can be challenging and is often accomplished using rare-metal catalysts with chiral ligands the syntheses of which are often not benign. On the other hand, enzymes are highly chemo-, regio-, and stereoselective and can readily be produced from biorenewable ingredients, while producing biodegradable waste.^1^ Developing a retrosynthesis of the chiral fragment **5** (Scheme 1B), asymmetric keto-reduction catalyzed by alcohol dehydrogenases (ADHs) is well established and implemented at industrial scale and would allow access to **5** from intermediate **4**.^1^ The chiral carbonitrile center in **4** may be generated by asymmetric reduction of the corresponding α,β-unsaturated ketone **3**, and while ene reductases (ERs) are attractive catalysts for this step, their implementation suffers from a lack of scalability.^3−5^ GlaxoSmithKline (GSK) recently described other approaches to 3-oxocyclohexane-1-carbonitrile **4**;^6^ however, these assume access to the precursor, and the kinetic resolution by nitrilases precludes full conversion.

Intermediate **3** not being commercially available, a C–H oxyfunctionalization step is required to introduce the ketone functional group into the commercially available pro-chiral starting material **1**. Regioselective allylic oxidation of alkenes, in particular substituted cyclohexenes, remains challenging^7^ and reported chemical strategies include the use of hypervalent iodide reagents,^8^ transition metal catalysis,^9,10^ or photocatalysis.^11,12^ Enzymatic strategies are underdeveloped;^13^ examples on few selected substrates include the use of cytochrome P450s and unspecific peroxygenases (UPOs).^14,15^ UPOs are well known to selectively catalyze the oxyfunctionalization of ethylbenzene at the benzylic position to the corresponding enantiopure (*R*)-1-phenylethanol, and can also further oxidize both enantiomers of 1-phenylethanol to acetophenone,^16^ yet remain to be further explored with substituted cyclohexene substrates, in particular with regard to stereoselectivity.^14,17^

Thus, we report an alternative enzymatic synthesis (Scheme 1C) of (1*S*,3*R*)-3-hydroxycyclohexanecarbonitrile **5** starting from the commercially available pro-chiral cyclohexene-1-nitrile **1**. The first step, a regioselective allylic oxidation, was enabled by a UPO after several chemical strategies proved unsuccessful. ER and ADH were then employed to generate the two chiral centers, and the cascade was carried out in a three-step one-pot system.

## Results and Discussion

**Figure 1:**
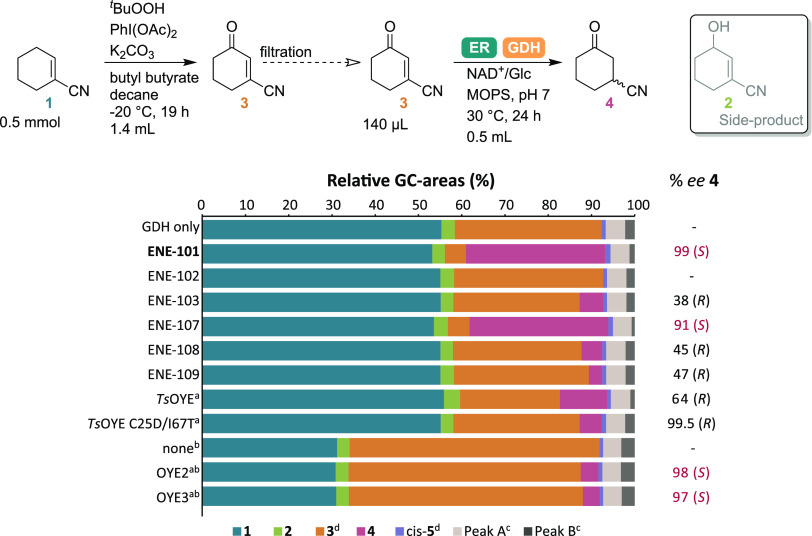
Chemical allylic oxidation of cyclohexene-1-nitrile **1**, followed by screening of a panel of ERs for the reduction of 3-oxocyclohex-1-ene-1-carbonitrile **3** to 3-oxocyclohexane-1-carbonitrile **4**. Analysis by GC-FID followed extraction with EtOAc on an Agilent CP-Sil 8 CB column for conversion and a Hydrodex β-6TBDM column for *ee*. Conditions: allylic oxidation: ^*t*^BuOOH (4–5 equiv), PhI(OAc)_2_ (1.5 equiv), K_2_CO_3_ (0.5 equiv), butyl butyrate (1 mL), decane (0.4 mL), −20 °C, 19 h. ER step: Filtered reaction mixture from previous step (140 μL), ER (2 mg/mL), GDH-101 (1 mg/mL), NAD^+^ (1 mol %), D-Glc (1.1 equiv), MOPS-NaOH (200 mM), pH 7, 0.5 mL, 30 °C, 900 rpm, 24 h. ^a^ purified enzyme, 0.2 mg/mL protein content; ^b^ −15 °C, 24 h allylic oxidation; ^c^ unidentified peaks; ^d^ overlapping with an unidentified impurity in ^*t*^BuOOH. Data are also shown in Table S1.

The choice of a suitable ER was the first step as few ERs are known to reduce β-substituted unsaturated substrates with enantiopurity.^18^ The allylic oxidation of cyclohexene-1-nitrile **1** followed by alkene reduction were investigated using a telescoped approach, as intermediate 3-oxocyclohex-1-ene-1-carbonitrile **3** was not commercially available. Thus, the unsaturated ketone **3** was generated by chemical treatment of **1** using ^*t*^BuOOH/PhI(OAc)_2_ in butyl butyrate (see the Supporting Information),^8^ which was then added (after filtration) to a buffered aqueous layer containing the components required for the second biocatalytic step (Figure 1). Using this approach, a panel of 11 ERs was screened, comprising in-house enzymes *Ts*OYE, *Ts*OYE C25D/I67T, OYE2, and OYE3 previously recombinantly produced in *Escherichia coli* on a 1 L scale and purified by affinity chromatography (see the Supporting Information), and the C=C-bond reduction panel from Johnson Matthey (JM), ENE-101 to -103 and -107 to -109 received as lyophilized cell-free extracts (Figure 1).^19^ Two ERs, ENE-101 and -107, showed high (*S*)-selectivity and high conversions from **3** to **4**. ENE-101 having the higher stereoselectivity at 99% *ee* of (*S*)-**4** was chosen for the cascade. Absolute stereochemistry was inferred from the known selectivities of *Ts*OYE C25D/I67T,^20^ OYE2, and OYE3.^21^

From this initial screening, chemical oxidation of **1**–**3** showed poor conversions (30–40%), unidentified side-products, and a lack of mass balance probably due to radical oligomerization (Figure S1). Investigating several alternative chemical allylic oxidation strategies, such as Rh_2_(cap)_4_-,^9^ or Mn(III)OAc-catalysis,^10^ or photocatalysis,^11,12^ was either unsuccessful, not scalable to the targeted substrate loading of 200 mM, or showed similar lack of mass balance (results shown in Figure S2). Therefore, a more efficient and reliable synthetic method for the selective allylic oxidation of substituted cyclohexenes is clearly needed.

**Figure 2:**
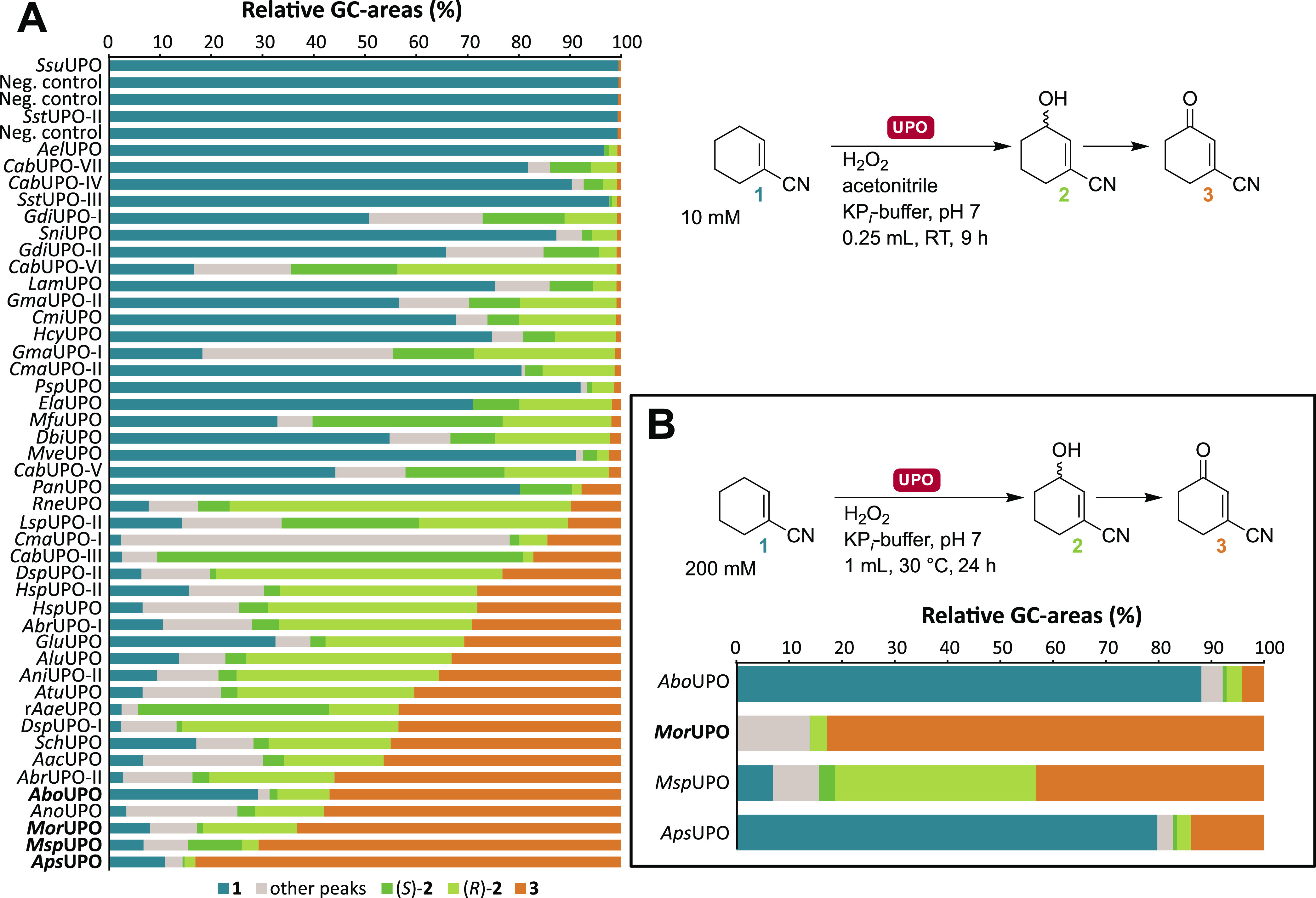
Screening of a panel of UPOs for the allylic oxidation of cyclohexene-1-nitrile **1**, sorted by relative amounts of the desired product **3**. **A**: initial screening at the 10 mM scale. Conditions: UPO (4–10 mg/mL), H_2_O_2_ (10 × 0.24 equiv), acetonitrile (5% *v/v*), KP_*i*_-buffer (100 mM), pH 7, 0.25 mL, RT, 600 rpm, 9 h in 96-deepwell plate. Negative controls are based on different expression strains without overexpressed UPO. **B**: Rescreening of top variants at targeted reaction scale. Conditions: UPO (10 mg/mL), H_2_O_2_ (0.1 eq./h), KP_*i*_-buffer (300 mM), pH 7, 1 mL, 30 °C, 600 rpm, 24 h. Concentrations are given with respect to the final reaction volume. Analysis by GC-FID following extraction with EtOAc on a Hydrodex β-6TBDM column for conversion and *ee*. Data are also shown in Table S2.

The use of unspecific peroxygenase (UPO) was tried with the variant PaDa-1 from *Agrocybe aegerita*, r*Aae*UPO,^15^ which was recently produced on a pilot scale by Hollmann and co-workers.^22^ Simply using hydrogen peroxide as the oxidant, catalytic amounts of r*Aae*UPO enabled the allylic oxidation at the desired scale with good mass balance, regio- and chemo-selectivity, albeit with a maximum yield of 50% for the unsaturated ketone **3** (Figures 2 and S3). Investigating the stereochemistry of the r*Aae*UPO-catalyzed reaction over time revealed the nonstereoselective allylic hydroxylation of **1** to **2**, followed by stereoselective overoxidation of (*R*)-**2** to **3**, leaving the remaining (*S*)-enantiomer unreacted, limiting the yield of **3** obtained with r*Aae*UPO to 50% (Figure S3). The catalytic activity of other redox enzymes that may be present to reduce the ketone is excluded due to the absence of a stoichiometric amount of a reductant.^16^ This unexpected enantioselectivity by r*Aae*UPO for overoxidation is opposite to the more commonly observed highly enantioselective oxyfunctionalization of ethylbenzene to (*R*)-1-phenylethanol, followed by nonselective overoxidation to acetophenone.^16^

To further investigate the peroxygenase-catalyzed allylic hydroxylation and overoxidation, a panel of 44 UPOs from Aminoverse was screened (Figure 2A).^23^ These UPOs were recently discovered and produced in *Pichia pastoris*.^23^ Very diverse results were obtained. First, several UPOs (e.g., *Cab*UPO-VI) showed high levels of oxyfunctionalization, but little overoxidation, while others favored overoxidation (e.g., *Aps*UPO). Interestingly, the enantiomeric excess of the remaining alcohol **2** was also diverse with examples of racemic (e.g., *Lsp*UPO-II), highly (*R*)-enriched (e.g., *Dsp*UPO-II), and highly (*S*)-enriched (e.g., *Cab*UPO-III) **2** being found. Lastly, several UPOs (e.g., *Cma*UPO-I) favored the formation of alternative, unidentified product(s) (possibly epoxide, regioisomers, or dioxyfunctionalized products). This highlights the potential for UPOs to selectively catalyze diverse allylic hydroxylation and oxidation reactions that are otherwise difficult to carry out.

For the purposes of the cascade, UPOs catalyzing high levels of overoxidation for both enantiomers (*S*)- and (*R*)-**2** were desired; however, the discovery of this enantiomeric overoxidation opens possibilities and should be further investigated. Thus, *Aps*UPO, *Msp*UPO, *Mor*UPO, and *Abo*UPO were chosen to be rescreened under the targeted reaction conditions, at 200 mM substrate loading and without any addition of organic cosolvents (Figure 2B). However, the performance of the enzymes changed when the substrate concentration and reaction conditions were modified. *Aps*UPO, which was the best-performing enzyme during the initial screen, showed very low levels of product formation at the reaction scale. On the other hand, *Mor*UPO showed very high levels of overoxidation under the reaction conditions and was thus chosen to be implemented in the cascade. Interestingly, *Mor*UPO was also recently reported to provide racemic 1-phenylethanol from ethylbenzene.^23^

**Figure 3:**
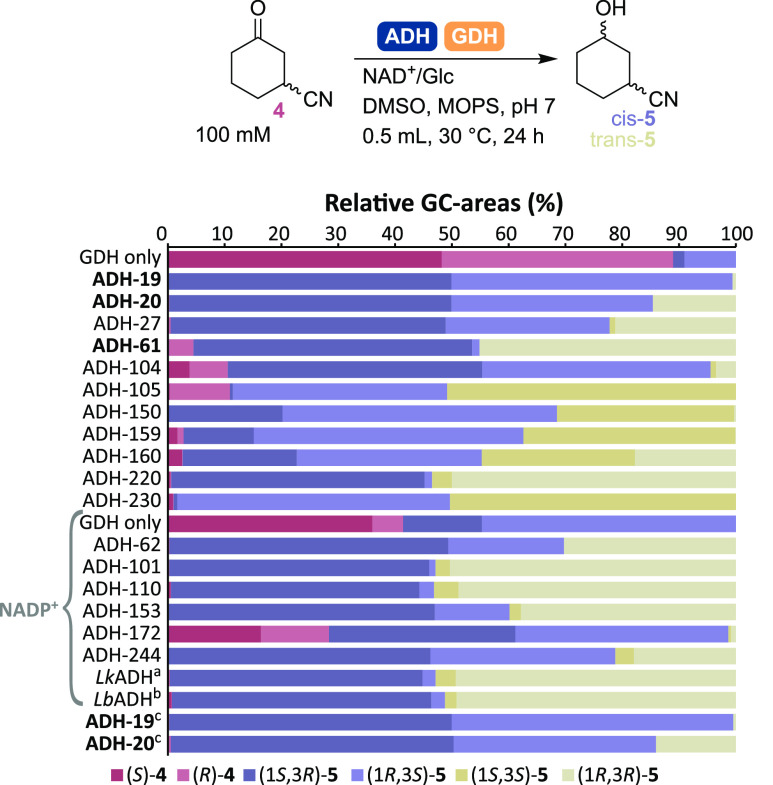
Screening of a panel of ADHs for the reduction of 3-oxocyclohexane-1-carbonitrile **4** to chiral 3-hydroxycyclohexanecarbonitrile **5**. Analyses by GC-FID following extraction with EtOAc on a Hydrodex β-6TBDM column for conversion and *ee*. Conditions: ADH (4 mg/mL), GDH-101 (2 mg/mL), NAD(P)^+^ (1 mol %), D-Glc (1.1 equiv), DMSO (10% *v/v*), MOPS (200 mM), pH 7, 0.5 mL, 30 °C, 900 rpm, 24 h. ^a^ cell-free extract, 4 mg/mL protein content; ^b^ purified enzyme, 0.4 mg/mL protein content; ^c^ ADH (2 mg/mL), and GDH-101 (1 mg/mL). Data are also shown in Table S3.

Next, a panel of ADHs was screened using racemic **4**, including in-house enzymes *Lb*ADH and *Lk*ADH, and the C=O-reduction kit provided by JM (Figure 3). Almost all ADHs screened achieved >90% conversion, accepting both enantiomers of **4**. Absolute stereochemistry at the hydroxy position has been inferred from the enantiomeric enrichment of the remaining starting material and comparison with chemical keto-reduction using sodium borohydride, which is known to be *cis*-selective (Figures S9–S12).^24−26^ ADH-19, -20, -61, and -62 as well as GDH-101 (or an impurity therein) exclusively produce the desired (1*S*,3*R*)-**5** from (*S*)-**4** but have varying selectivities with (*R*)-**4**. ADH-19, -20, and GDH-101 preferentially produce the *cis*-diastereomer, while ADH-61 and -62 are (*R*)-selective, albeit with lower selectivity when using (*R*)-**4** as opposed to (*S*)-**4**. Compared with the results with the ADHs, the GDH-catalyzed reaction is much slower and is probably not synthetically useful. Given the high enantioselectivity of ER-101, all four ADHs may be suitable enzymes for the desired cascade; however, ADH-62 was omitted from the further development of the cascade due to its dependence on NADP^+^, which has a higher cost than NAD^+^.

Suitable catalysts having been identified for each of the steps of the cascade, we set out to integrate the separate steps into a one-pot three-step setup. Initial attempts with the original allylic oxidation using the ^*t*^BuOOH/PhI(OAc)_2_ system revealed that ADH-61 was not suitable (Figure S4A), while attempts with r*Aae*UPO showed that ADH-20 was slightly more robust than ADH-19 (Figure S4B). Combining the *Mor*UPO-catalyzed allylic oxidation with ENE-101-catalyzed C=C reduction, very low conversions were obtained for the ER step, while full conversions were achieved when r*Aae*UPO had been used (Figure S4C). Increasing the concentration of ENE-101 and the reaction time still resulted in low conversions for the ER step (Figure S4C). Attempts to carry out the UPO and ER reactions concurrently were unsuccesul (data not shown), potentially due to the oxidation of the alcohol groups in glucose by UPO, although such products are not detectable by GC. Overall, the cascade had to be run in a one-pot three-step approach, due to the incompatibility of UPO with the cofactor recycling system,^16^ and the ADH-catalyzed reaction being sufficiently fast (relative to the ER) to produce significant quantities of the unsaturated alcohol intermediate **2**, which is not a substrate for the ER (data not shown).

The key to combining UPO and ER as a one-pot two-step cascade was that the substrate concentration had to be decreased. The ER step had shown good conversion with r*Aae*UPO, which only gave 50% yield of the intermediate **3**, unlike *Mor*UPO, which overoxidized both hydroxylated enantiomers (*S*)- and (*R*)-**2**. Indeed, decreasing the targeted final concentration by half to 50 mM (thus matching concentrations of **3** achieved with r*Aae*UPO), full conversion of **3** to **4** was achieved for the ER step (Figure S4C).

**Figure 4:**
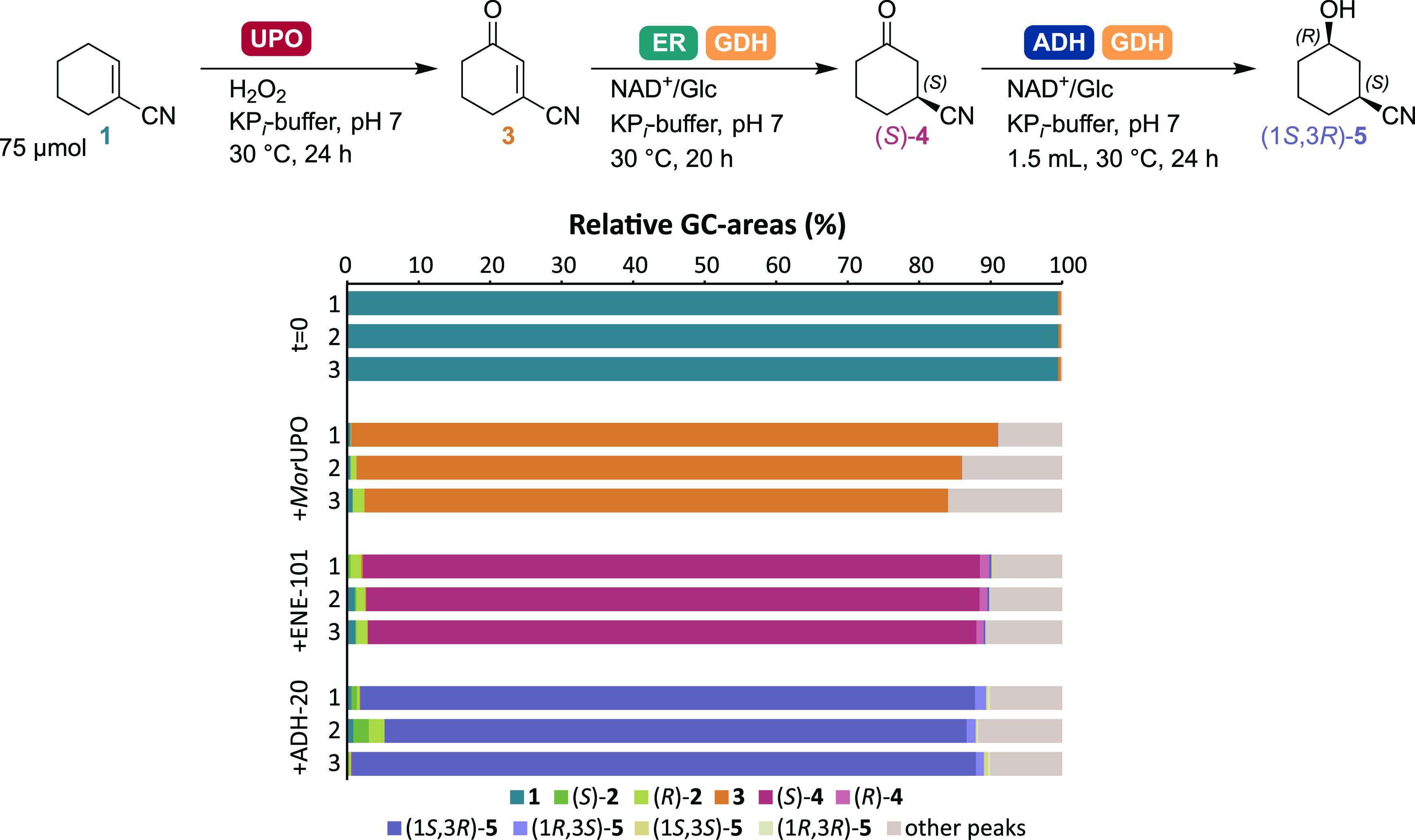
Full cascade from **1** to **5**, using *Mor*UPO, ENE-101, ADH-20. A total of 12 reactions were set up and sets of three were quenched and analyzed at the start of the cascade, and after each of the three enzymatic steps. Analyses by GC-FID following extraction with EtOAc on a Hydrodex β-6TBDM column for conversion and *ee*. Conditions: UPO-step: *Mor*UPO (3.75 mg/mL), H_2_O_2_ (0.1 equiv/h for 22 h), KP_*i*_-buffer (300 mM), pH 7, 30 °C, 600 rpm, 24 h, final volume 990 μL. ER step: addition of ER (3 mg), GDH-101 (1.5 mg), NAD^+^ (2 mol %), D-Glc (1.1 equiv), 30 °C, 900 rpm, 20 h, final volume 1245 μL. ADH step: addition of ADH (3 mg), GDH-101 (1.5 mg), NAD^+^ (2 mol %), D-Glc (1.1 equiv), 30 °C, 900 rpm, 24 h, final volume 1500 μL. Data are also shown in Table S4.

Having solved the first two steps, the third ADH step was introduced into the final cascade. Extraction with ethyl acetate provided the final product, reaching product titers of 85% (corresponding to 5.3 g/L) of (1*S*,3*R*)-**5** in three replicates of the cascade (Figure 4), with 97% *ee* and 99% *de*, on average. When directly extracted in deuterated chloroform (without further purification), NMR analysis of the product showed a relatively clean spectrum and was consistent with the expected *cis*-stereoisomer, further confirming the stereochemical assignment (Figures S16–S18).^27^ This one-pot three-step UPO-ER-ADH multienzymatic system therefore provides access to an oxyfunctionalized chiral API with two stereocenters with high purity and enantiomeric excess.

## Conclusions

From a biocatalytic retrosynthetic analysis, a cosolvent-free one-pot three-step cascade was developed for the synthesis of a chiral building block of the LPA_1_-Antagonist BMS-986278. The key to the development of the cascade was the unprecedented peroxygenase-catalyzed allylic oxidation of a substituted cyclohexene and screening of panels of UPOs, ERs, and ADHs to identify suitable enzymes. The overall cascade could be carried out at 50 mM scale (final concentration) without cosolvent, the limitation being the ER step. Enzyme loadings were 47 wt % for *Mor*UPO and 37 wt % for the other enzymes, with respect to **1**. Both the substrate concentration and enzyme loading can be dramatically improved by directed evolution, as has been frequently demonstrated by industry.^28,29^ GDH-101 showed promiscuous ADH activity; however, as it produced the desired stereochemical outcome from (*S*)-**4**, this was not interfering with the cascade but would require further investigation to access the *trans*-diastereomer with high selectivity. The promiscuity could be attenuated by using NAD^+^ rather than NADP^+^.

The unreported stereochemical behavior of the frequently used r*Aae*UPO was observed, in which oxyfunctionalization was not stereoselective, yet overoxidation was highly enantioselective. This surprising stereoselectivity was overcome by using *Mor*UPO, albeit with an increase in unidentified side-products (likely other regioisomers, epoxide, or dioxyfunctionalized compounds), leaving room for further improvement via enzyme discovery or protein engineering of UPOs.

## Supporting Information Available

Materials and methods; time course of the chemical allylic oxidation of cyclohexene-1-nitrile **1** (Figure S1); different chemical allylic oxidation strategies attempted (Figure S2); time course of the allylic oxidation (Figure S3); early attempts at the combined cascade (Figure S4); SDS-PAGE gels (Figure S5); chemical allylic oxidation of cyclohexene-1-nitrile **1** and ER screening (Table S1); UPO screening (Table S2); ADH screening (Table S3); full cascade from **1** to **5** (Table S4); stacked GC chromatograms (Figures S6 and S7); chiral GC chromatograms (Figures S8–S15); ^1^H-NMR spectrum (Figure S16); ^13^C-NMR spectrum (Figure S17); and multiplicity edited ^1^H-^13^C-HSQC spectrum (Figure S18) (PDF)
