# Supplementary material for: Peroxygenase-Catalyzed Allylic Oxidation Unlocks Telescoped Synthesis of (1S,3R)-3-Hydroxycyclohexanecarbonitrile
Source: ACS Catal. 2024 Feb 13;14(5):2985–91. doi: 10.1021/acscatal.4c00177 (PMC10913032; doi:10.1021/acscatal.4c00177)
Supplement: Supplementary file 1 — cs4c00177_si_001.pdf [file cs4c00177_si_001.pdf]

Electronic Supporting Information

Peroxygenase-catalyzed allylic oxidation unlocks telescoped synthesis of (1*S*,3*R*)-3-hydroxycyclohexanecarbonitrile

Christian M. Heckmann,[a] Moritz Bürgler,[b] Caroline E. Paul[a,]*

[a] *Biocatalysis section, Department of Biotechnology, Delft University of Technology, van der Maasweg 9, 2629HZ Delft, The Netherlands*
[b] *Bisy GmbH, Wünschendorf 292, 8200 Hofstätten an der Raab, Austria*

Contents

Materials and Methods 2
Allylic oxidation using $PhI(OAc)_2$ and ^tBuOOH 2
Screening of ERs 2
Screening of ADHs 2
Screening of UPOs 2
Combined cascade 3
Analytical methods 3
Supplementary Figures 5
Supplementary Tables 8
GC chromatograms 11
NMR spectra 16
References 18

Materials and Methods

Chemicals were purchased from Sigma Aldrich. $NAD(P)^+$, ADHs, ERs, and GDH-101 were kindly provided by Johnson Matthey in the form of lyophilized cell-free extracts (CFEs). The panel of UPOs (produced by recombinant expression in *Pichia pastoris*)[1] was purchased from Aminoverse. *Lb*ADH, *Lk*ADH, *Ts*OYE, *Ts*OYE C25D/I67T, OYE2, OYE3 and *Aae*UPO PaDa-I (r*Aae*UPO)[2] were used from frozen stock solutions or lyophilized powders available from previous work.[3,4]

Allylic oxidation using PhI(OAc)$_2$ and ^{t}BuOOH

Based on a previous reference:[5] To (diacetoxyiodo)benzene (484 mg, 1.50 mmol) and potassium carbonate (35 mg, 0.25 mmol) was added *n*-butyl *n*-butanoate (1 mL) and the mixture was cooled to -20 or -15 °C, after which cyclohex-1-enecarbonitrile **1** (56.6 µL, 0.5 mmol) was added. With vigorous stirring, *t*-butyl hydroperoxide (400 µL of 5-6 M solution in decane) was added slowly over 30 min. The reaction was stirred at -20 or -15 °C for 24 h and filtered through a cotton plug prior to use in the ER screening or dilution in EtOAc for GC analysis (see analytical methods) or $CDCl_3$ for NMR analysis.

Screening of ERs

Biotransformations (0.5 mL final volume) were carried out in MOPS-buffer (200 mM, pH 7.0) by adding the following (from 10-fold concentrated stock solutions): NAD^+ (1 mol%), D-glucose (D-Glc, 1.1 eq.), GDH-101 (1 mg/mL lyophilized CFE), ER (JM enzymes: 2 mg/mL lyophilized CFE; *Ts*OYE, *Ts*OYE C25D/I67T, OYE2, and OYE3: 0.2 mg/mL purified enzyme). Lastly, the allylic oxidation reaction mixture (140 µL; approx. 50 µmol) was added and the biotransformations were incubated at 30 °C, 900 rpm (Eppendorf Thermomixer C) for 24 h. Reactions were extracted with EtOAc (1 mL). The extract was diluted 2-fold with EtOAc, and analysed for conversion and enantiomeric excess (after drying with sodium sulfate Na_2SO_4) as described under analytical methods.

Screening of ADHs

Biotransformations (0.5 mL final volume) were carried out in MOPS-buffer (200 mM, pH 7.0) by adding the following from 10-fold concentrated stock solutions in buffer: D-Glc (1.1 eq.), $NAD(P)^+$ (1 mol%), racemic 3-cyanocyclohexanone **4** (50 µmol, in DMSO (final concentration 10% *v/v*)), GDH-101 (2 mg/mL lyophilized CFE), and ADH (JM enzymes: 2 mg/mL lyophilized CFE; *Lb*ADH: 0.4 mg/mL purified enzyme; *Lk*ADH: 4 mg/mL protein content CFE). Biotransformations were incubated at 30 °C, 900 rpm (Eppendorf Thermomixer C) for 24 h. Reactions were extracted with EtOAc (1 mL). The extract was diluted 2-fold with EtOAc, and analysed for conversion and enantiomeric excess (after drying with Na_2SO_4) as described under analytical methods.

NAD^+ was used for all ADHs except the for following $NADP^+$-dependent enzymes: ADH-62,-101,-110,-153,-171, -244, *Lb*ADH, and *Lk*ADH.

Screening of UPOs

One replicate of the enzymes provided in the UPO enzyme panel purchased from Aminoverse, as well as lyophilized r*Aae*UPO (2-5 mg) were resuspended in MilliQ H_2O (100 µL). Reactions were assembled in a 96-deep-weel-plate (DWP) by sequentially adding potassium phosphate buffer (KP_i-buffer 100 mM, pH 7.0; 97.5 µL), resuspended UPO (50 µL), and substrate **1** (200 mM in acetonitrile; 12.5 µL). Hydrogen peroxide (66.7 mM; 10 µL) was added once per hour over the course of 9 h. The DWP was sealed with an

aluminium seal and incubated at ambient temperature (20 °C) with shaking (600 rpm) in between hydrogen peroxide additions. After 9 h, EtOAc (1 mL) was added to each well and each well was transferred to a microfuge tube for extraction. The EtOAc supernatant was dried with Na_2SO_4, and analysed by chiral GC-FID as described under analytical methods (split ratio 10:1).

Combined cascade

Biotransformations (final volume 1.5 mL) were carried out in KP_i-buffer (300 mM, pH 7.0) by combining buffer (454 µL), cyclohex-1-enecarbonitrile **1** (8.50 µL, 75 µmol), and *Mor*UPO (150 µL of 25 mg/mL stock). The reaction was incubated at 30 °C, 600 rpm (Eppendorf Thermomixer C) for 24 h with hydrogen peroxide being fed via syringe pump (17.1 µL/h of a 438 mM stock, corresponding to 7.5 µmol/h) for the first 22 h. D-Glc (75 µL of 1.1 M stock), NAD^+ (30 µL of 50 mM stock), GDH-101 (75 µL of a 20 mg/mL stock), and ENE-101 (75 µL of a 40 mg/mL stock) were added, and the reaction incubated at 30 °C, 900 rpm for a further 20 h. D-Glc (75 µL of 1.1 M stock), NAD^+ (30 µL of 50 mM stock), GDH-101 (75 µL of a 20 mg/mL stock), and ADH-20 (75 µL of a 40 mg/mL stock) were added, and the reaction incubated at 30 °C, 900 rpm for a further 24 h. Reactions were extracted with EtOAc (1 mL). The extract was diluted 5-fold with EtOAc and analysed for conversion and enantiomeric excess (after drying with Na_2SO_4) as described under analytical methods. Alternatively, the reaction was extracted with $CDCl_3$ and analysed by ^{1}H- and ^{13}C-NMR. ^{1}H-NMR (400 MHz, $CDCl_3$, referenced relative to TMS) δ 1.23–1.39 (m, 2H), 1.43–1.60 (m, 2H), 1.86–1.93 (m, 1H), 1.94–2.05 (m, 2H), 2.29 (dtt, *J* = 12.4, 3.8 Hz, 1.8 Hz, 1H), 2.50 (tt, *J* = 11.8, 3.7 Hz, 1 H), 3.61 (tt, *J* = 10.2, 4.2 Hz, 1 H); ^{13}C-NMR (101 MHz, $CDCl_3$, absolute referencing relative to ^{1}H-NMR) δ 22.7 (CH_2), 26.7 (CH), 28.9 (CH_2), 34.2 (CH_2), 37.8 (CH_2), 68.5 (CH), 121.7 (C). See NMR spectra page S16.

Analytical methods

The identity of product **5** was confirmed by chemical reduction of **3** with sodium borohydride $NaBH_4$: racemic **4** (20 µL), and $NaBH_4$ (20 mg) were added to methanol (1 mL), on ice. The mixture was brought to ambient temperature (20 °C) over 15 min. After addition of aqueous sodium hydroxide (0.1 M; 1 mL), the mixture was concentrated *in vacuo*, neutralized with hydrochloric acid and extracted with EtOAc (1 mL).

Conversions were determined by GC-FID on a GC-2010 Plus (Shimadzu, Japan) equipped with an AOC-20i auto injector and a flame ionization detector (FID), using an Agilent CP-Sil 8 CB column (25 m × 0.25 mm × 1.2 µm). 1 µL of sample was injected with a split ratio of 25:1 and injector temperature of 340 °C. The FID was maintained at 360 °C. Nitrogen was used as the carrier gas, with an initial linear velocity of 30 cm/s.

Oven temperature program:

Ramp (°C/min)	Temperature (°C)	Hold (min)
	80	1
10	200	1
30	345	1

Enantiomeric excess was determined by GC-FID on a GC-2010 Plus (Shimadzu, Japan) equipped with an AOC-20i auto injector and a flame ionization detector (FID), using a Hydrodex β-6TBDM column (50 m × 0.25 mm). Samples were dried using Na_2SO_4 prior to analysis. 1 µL of sample was injected with a split ratio of 50:1 and injector temperature of 250 °C. The FID was maintained at 275 °C. Helium was used as the carrier gas, with an initial linear velocity of 38 cm/s.

Oven temperature program:

Ramp (°C/min)	Temperature (°C)	Hold (min)
	130	1
5	175	10
25	245	1

To achieve improved separation between a UPO side-product peak and the different stereoisomers of **5**, the initial linear velocity was reduced to 30 cm/s and the temperature program was adapted as follows:

Ramp (°C/min)	Temperature (°C)	Hold (min)
	130	1
5	175	15
25	245	1

Supplementary Figures

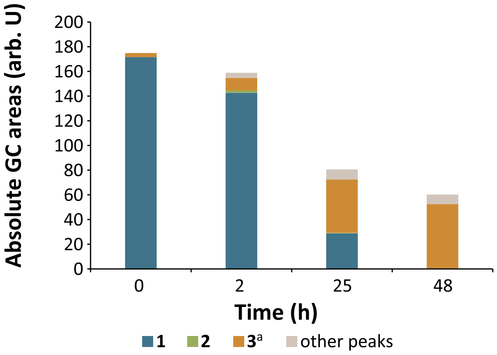

Figure S1. Time course of the chemical allylic oxidation of cyclohexene-1-nitrile **1** (using diacetoxyiodo)benzene and *t*-butyl hydroperoxide). Analysis by GC-FID following extraction with EtOAc on an Agilent CP-Sil 8 CB column for conversion. [a] overlapping with an unidentified impurity in ^{t}BuOOH.

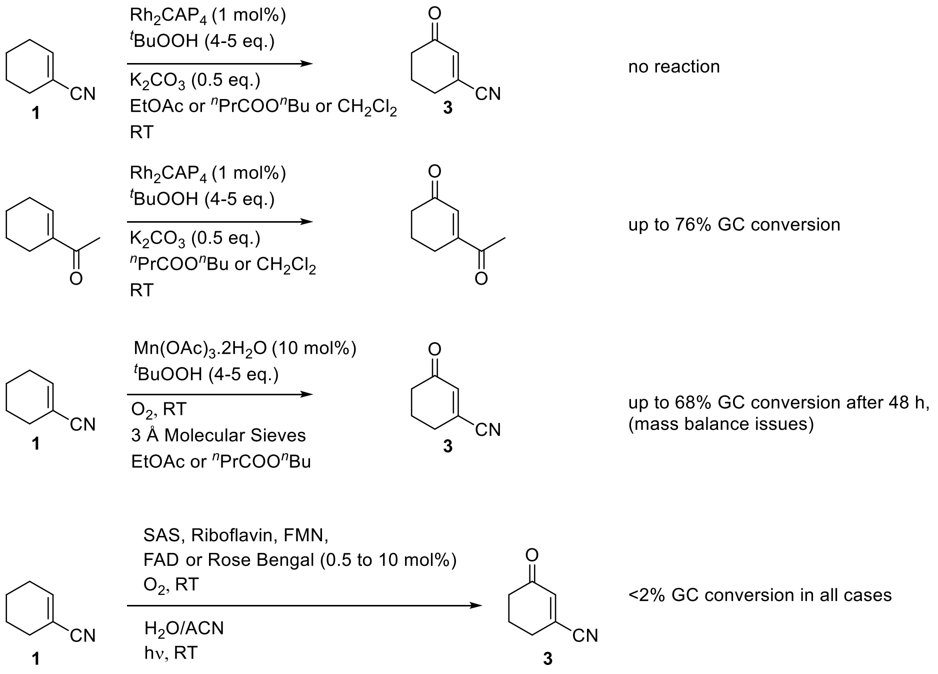

Figure S2. Different chemical allylic oxidation strategies attempted. Rh_2CAP_4 catalysed allylic oxidation[6] showed no conversion with the nitrile substituent, while up to 76% conversion was obtained with an acetyl substituent. Reactions with the nitrile substituent showed rapid decolourization of the red catalytically active Rh complex, indicating substrate-catalyst incompatibility. $Mn(OAc)_3$ catalysed allylic oxidation[7] showed GC conversions (relative peak areas) of 68% after 48 h, but absolute peak areas decreased similarly to the reaction using diacetoxyiodo)benzene. Various photocatalytic allylic oxidations were also attempted, all reaching less than 2% GC conversion. Anthraquinone sulfate (SAS)[8] has previously been reported with the methyl substituted cyclohexene, reaching product concentrations in neat substrate of 70 mM, with a regio-isomer ratio of 1.3:1. Riboflavin has been reported to facilitate benzylic oxidations with up to 58% conversion at 10 mM substrate loading in H_2O/MeCN.[9] Rose Bengal has previously been suggested to facilitate benzylic oxidations in acetonitrile.[10]

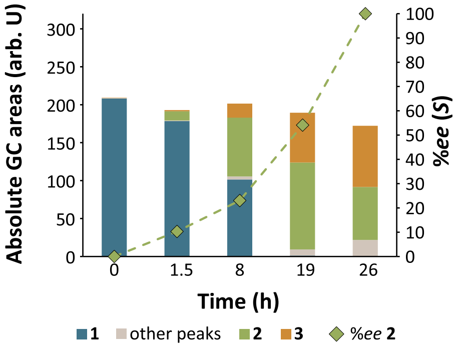

Figure S3. Time course of the allylic oxidation catalysed by r*Aae*UPO of cyclohexene-1-nitrile **1**. Analysis by GC-FID following extraction with EtOAc on an Agilent CP-Sil 8 CB column for conversion and Hydrodex β-6TBDM column for *ee*.

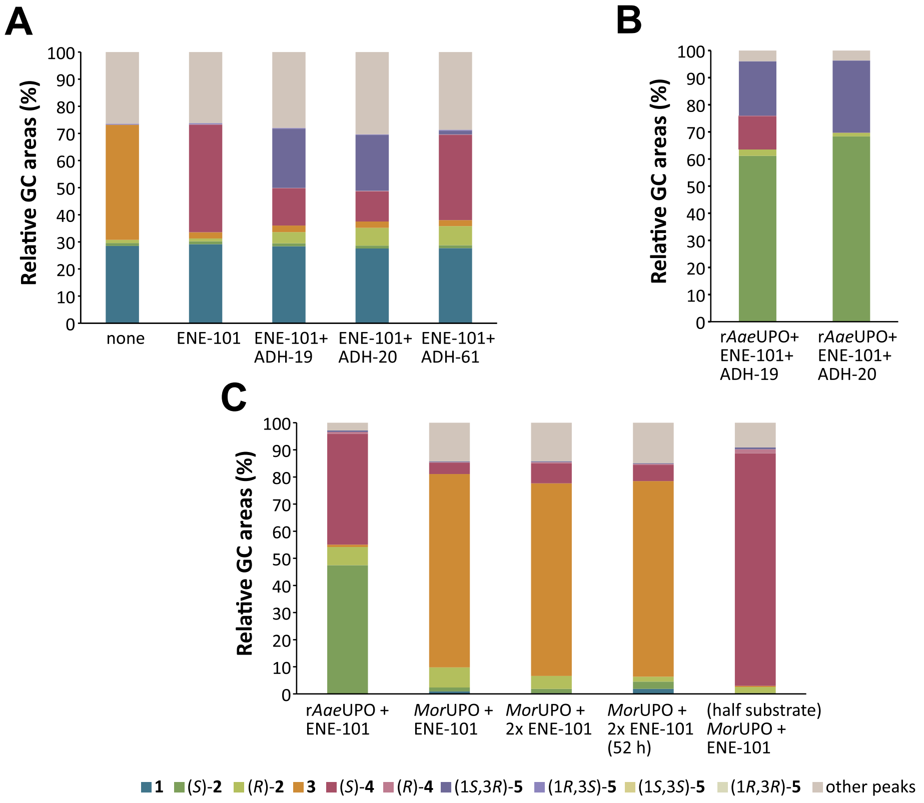

Figure S4. **A**: Combined cascade starting from the chemical allylic oxidation of cyclohexene-1-nitrile **1** (using diacetoxyiodo)benzene and *t*-butyl hydroperoxide). **B**: Fully enzymatic cascade with r*Aae*UPO and ENE-101, comparing ADH-19 and ADH-20 in the last step. **C**: Comparison of the ER step, starting from *r*AaeUPO or *Mor*UPO with varying conditions. Analysis by GC-FID following extraction with EtOAc on a Hydrodex β-6TBDM column for conversion and *ee*.

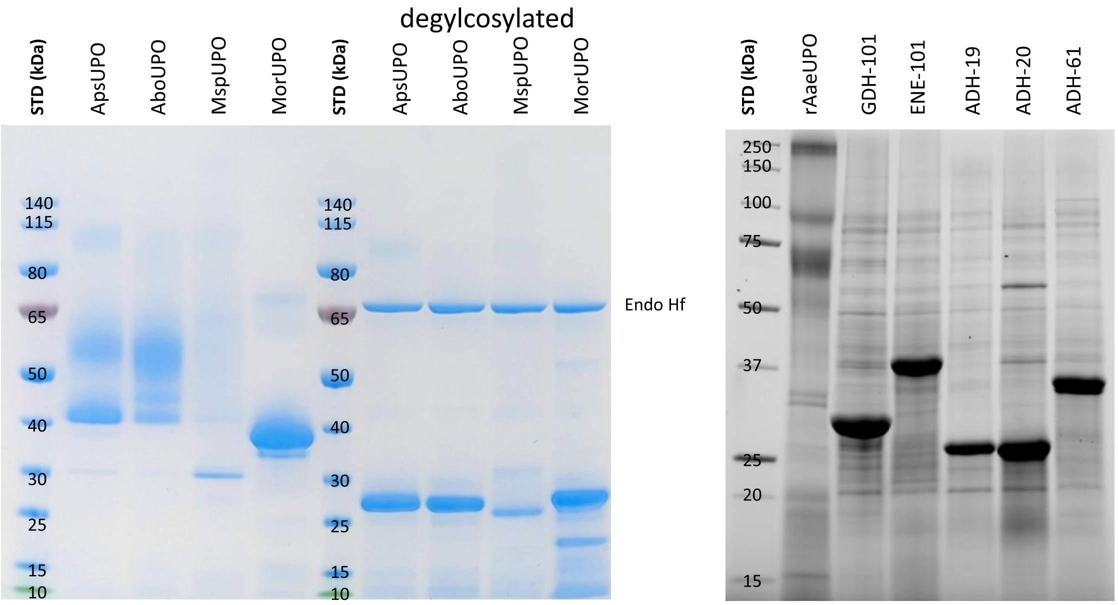

Figure S5. SDS-PAGE gels of the best performing enzymes. **Left**: best performing UPOs from the Aminoverse panel. Deglycosylation was performed with Endo Hf. **Right**: r*Aae*UPO, best performing lyophilized GDH, ERED and ADH from the JM panels.

Supplementary Tables

Table S1. Chemical allylic oxidation of cyclohexene-1-nitrile **1**, followed by screening of a panel of ERs for the reduction of 3-oxocyclohex-1-ene-1-carbonitrile **3** to 3-oxocyclohexane-1-carbonitrile **4**. Analysis by GC-FID following extraction with EtOAc on an Agilent CP-Sil 8 CB column for conversion and Hydrodex β-6TBDM column for *ee*.

ER	Relative GC areas (%)							
	**1**	**2**	**3** [d]	**4**	*cis*-**5**[d]	Peak A[c]	Peak B[c]	*ee* **4**
GDH only	55.3	3.1	33.9	0	1	4.5	2.2	-
**ENE-101**	**53.2**	**2.9**	**4.9**	**32.2**	**1.2**	**4.4**	**1.2**	**99.2**
ENE-102	55	3.2	34.4	0	0.9	4.5	1.9	-
ENE-103	55.1	2.9	29.2	5.4	0.9	4.5	1.9	-38.4
ENE-107	53.6	3.2	5	32.1	1.1	4.3	0.7	90.8
ENE-108	55.1	2.9	29.7	4.8	0.9	4.5	2.1	-45
ENE-109	55.1	3.1	31.2	3.1	0.9	4.5	2.1	-47
*Ts*OYE [a]	55.9	3.7	23.1	10.9	0.9	4.5	1	-63.6
*Ts*OYE C25D/I67T [a]	55.1	2.9	29.2	5.1	1	4.4	2.2	-99.6
GDH only [b]	31.2	2.9	57.8	0	0.8	4.2	3.1	-
OYE2 [ab]	30.9	3	53.8	4.1	0.9	4.3	3.2	98.2
OYE3 [ab]	31	2.9	54.1	3.9	0.8	4.3	3	97.4

Conditions: **allylic oxidation step:** ^tBuOOH (4-5 eq.), $PhI(OAc)_2$ (1.5 eq.), K_2CO_3 (0.5 eq.), butyl butyrate (1 mL), decane (0.4 mL), -20 °C, 19 h. **ER step:** Filtered reaction mixture from previous step (140 µL), ER (2 mg/mL), GDH-101 (1 mg/mL), NAD^+ (1 mol%), D-Glc (1.1 eq.), MOPS-NaOH (200 mM), pH 7, 0.5 mL, 30 °C, 900 rpm, 24 h.
[a] purified enzyme, 0.2 mg/mL protein content; [b] -15 °C, 24 h allylic oxidation; [c] unidentified peaks; [d] overlapping with an unidentified impurity in ^tBuOOH.

Table S2. Screening of a panel of UPOs for the allylic oxidation of cyclohexene-1-nitrile **1,** sorted by relative amounts of desired product **3**. Initial screening at 10 mM scale. Negative controls are based on different expression strains, without overexpressed UPO. Concentrations are with respect to the final reaction volume. Analysis by GC-FID following extraction with EtOAc on a Hydrodex β-6TBDM column for conversion and *ee*.

UPO	Relative GC areas (%)				
	**1**	(*S*)-**2**	(*R*)-**2**	**3**	Other peaks
SsuUPO	99.5	0.0	0.0	0.5	0.0
Neg. control	99.5	0.0	0.0	0.5	0.0
Neg. control	99.4	0.0	0.0	0.6	0.0
SstUPO-II	99.3	0.0	0.0	0.7	0.0
Neg. control	99.3	0.0	0.0	0.7	0.0
*Ael*UPO	96.7	0.9	1.6	0.7	0.0
*Cab*UPO-VII	81.8	8.0	5.1	0.8	4.3
*Cab*UPO-IV	90.4	3.8	2.8	0.8	2.3
*Sst*UPO-III	97.7	0.5	1.0	0.8	0.0
*Gdi*UPO-I	50.7	16.0	10.3	0.8	22.2
*Sni*UPO	87.4	2.0	4.9	0.8	5.0
*Gdi*UPO-II	65.8	10.8	3.4	0.9	19.1
*Cab*UPO-VI	16.6	20.8	42.7	0.9	18.9
*Lam*UPO	75.4	8.4	4.7	0.9	10.6
*Gma*UPO-II	56.7	9.9	18.8	0.9	13.6
*Cmi*UPO	67.8	6.2	19.0	0.9	6.1
*Hcy*UPO	74.8	6.2	12.0	1.0	6.1
*Gma*UPO-I	18.3	15.8	27.6	1.2	37.2
*Cma*UPO-II	80.6	3.5	14.1	1.3	0.6
*Psp*UPO	92.1	1.0	4.3	1.4	1.3
*Ela*UPO	71.1	9.1	18.1	1.8	0.0
*Mfu*UPO	32.9	37.1	21.2	1.9	6.8

*Dbi*UPO	54.8	8.7	22.5	2.1	11.9
*Mve*UPO	91.2	2.7	2.4	2.3	1.3
CabUPO-V	44.2	19.3	20.3	2.4	13.7
*Pan*UPO	80.2	10.2	1.9	7.7	0.0
*Rne*UPO	7.8	6.2	66.6	9.8	9.6
Lsp-UPO-II	14.3	26.8	29.1	10.4	19.4
*Cma*UPO-I	2.4	1.9	5.5	14.4	75.9
*Cab*UPO-III	2.6	71.5	1.9	17.2	6.9
*Dsp*UPO-II	6.4	1.2	55.9	23.2	13.4
*Hsp*UPO-II	15.6	3.1	38.5	28.1	14.7
*Hsp*UPO	6.6	5.6	40.9	28.1	18.8
*Abr*UPO-I	10.6	5.3	37.6	29.2	17.4
*Glu*UPO	32.5	2.9	27.1	30.6	6.8
*Alu*UPO	13.7	4.1	40.0	33.1	9.0
*Ani*UPO-II	9.4	3.5	39.5	35.5	12.0
*Atu*UPO	6.6	3.2	34.5	40.4	15.3
r*Aae*UPO	2.5	37.4	13.5	43.4	3.2
*Dsp*UPO-I	2.4	1.1	42.2	43.5	10.8
*Sch*UPO	17.1	2.9	23.8	45.0	11.2
*Aac*UPO	6.7	4.0	19.5	46.4	23.4
*Abr*UPO-II	2.7	3.3	24.4	56.0	13.6
***Abo*UPO**	29.1	1.6	10.1	56.9	2.2
*Ano*UPO	3.4	3.4	13.4	58.0	21.7
***Mor*UPO**	8.0	1.2	18.4	63.2	9.2
***Msp*UPO**	6.7	10.6	3.3	70.8	8.6
***Aps*UPO**	10.9	0.4	2.1	83.1	3.5
***Abo*UPO** [a]	88.1	0.8	2.9	4.1	4.0
***Mor*UPO** [a]	0.1	0.2	3.2	82.8	13.8
***Msp*UPO** [a]	6.9	3.1	38.1	43.1	8.7
***Aps*UPO** [a]	79.8	0.8	2.6	13.9	3.0

Initial screening at 10 mM scale. Conditions: UPO (4-10 mg/mL), H_2O_2 (10 × 0.24 eq.), acetonitrile (5% *v/v*), KP_i-buffer (100 mM), pH 7.0, 0.25 mL, RT (20 °C), 600 rpm, 9 h in 96-deepwell plate.

[a] Re-screening of top variants at targeted reaction scale. Conditions: UPO (10 mg/mL), H_2O_2 (0.1 eq./h), KP_i-buffer (300 mM), pH 7, 1 mL, 30 °C, 600 rpm, 24 h.

Table S3. Screening of a panel of ADHs for the reduction of 3-oxocyclohexane-1-carbonitrile **4** to chiral 3-hydroxycyclohexanecarbonitrile **5**. Analyses by GC-FID following extraction with EtOAc on a Hydrodex β-6TBDM column for conversion and *ee*.

ADH	Relative GC areas (%)					
	(*S*)-**4**	(*R*)-**4**	(1*S*,3*R*)-**5**	(1*R*,3*S*)-**5**	(1*S*,3*S*)-**5**	(1*R*,3*R*)-**5**
GDH only	54.2	45.8	18.1	81.9	0	0
**19**	44.2	55.8	**49.8**	**49.6**	**0**	**0.6**
**20**	32.5	67.5	**49.8**	**35.5**	**0**	**14.7**
27	23.3	76.7	48.7	29	1	21.3
**61**	2	98	**51.4**	**1.4**	**0**	**47.3**
104	36.4	63.6	50.1	44.9	1.1	3.9
105	2.7	97.3	0.6	42.3	57.1	0
150	53	47	20.1	48.4	31.2	0.3
159	61.2	38.8	12.7	48.8	38.4	0.1
160	96.7	3.3	20.6	33.4	27.7	18.3
220	80.3	19.7	44.9	1.3	3.5	50.3
230	91.2	8.8	0.7	48.5	50.8	0
GDH only	*86.9*	*13.1*	*23.7*	*76.3*	*0*	*0*
62	*34.5*	*65.5*	*49.2*	*20.4*	*0*	*30.3*
101	*78.6*	*21.4*	*45.9*	*1.1*	*2.5*	*50.5*
110	*89.7*	*10.3*	*44*	*2.6*	*4.3*	*49.1*
153	*44.7*	*55.3*	*46.9*	*13.2*	*2*	*37.9*
172	*57.8*	*42.2*	*45.8*	*52.4*	*0.7*	*1.2*
244	*53*	*47*	*46.1*	*32.6*	*3.3*	*18*
LkADH[a]	*73.2*	*26.8*	*44.7*	*2.3*	*3.5*	*49.5*
LbADH[b]	*90.6*	*9.4*	*46*	*2.4*	*2.1*	*49.5*
**19**[c]	**35.3**	**64.7**	**49.7**	**49.9**	**0**	**0.5**
**20**[c]	**17.8**	**82.2**	**35.8**	**50.1**	**0**	**14.1**

Conditions: ADH (4 mg/mL), GDH-101 (2 mg/mL), NAD^+ (1 mol%), D-Glc (1.1 eq.), DMSO (10% *v/v*), MOPS-NaOH (200 mM), pH 7.0, 0.5 mL, 30 °C, 900 rpm, 24 h. Entries in italics use $NADP^+$ instead of NAD^+.
[a] CFE, 4 mg/mL protein content; [b] purified enzyme, 0.4 mg/mL protein content; [c] ADH (2 mg/mL), GDH-101 (1 mg/mL).

Table S4. The full cascade from **1** to **5**, using *Mor*UPO, ENE-101, ADH-20. A total of 12 reactions were carried out, and sets of three were quenched and analyzed at the start of the cascade, and after each of the three enzymatic steps. Analyses by GC-FID following extraction with EtOAc on a Hydrodex β-6TBDM column for conversion and *ee*.

Progress		Relative GC areas (%)										
		**1**	(*S*)-**2**	(*R*)-**2**	**3**	(*S*)-**4**	(*R*)-**4**	(1*S*,3*R*)-**5**	(1*R*,3*S*)-**5**	(1*S*,3*S*)-**5**	(1*R*,3*R*)-**5**	other
T = 0	1	99.4	0.0	0.0	0.4	0.0	0.0	0.0	0.0	0.0	0.0	0.1
	2	99.5	0.0	0.0	0.4	0.0	0.0	0.0	0.0	0.0	0.0	0.1
	3	99.4	0.0	0.0	0.4	0.0	0.0	0.0	0.0	0.0	0.0	0.2
+ *Mor*UPO	1	0.5	0.0	0.1	90.4	0.0	0.0	0.0	0.0	0.0	0.0	8.9
	2	0.5	0.0	0.9	84.7	0.0	0.0	0.0	0.0	0.0	0.0	14.0
	3	0.8	0.0	1.6	81.6	0.0	0.0	0.0	0.0	0.0	0.0	16.0
+ ENE-101	1	0.0	0.5	1.5	0.2	86.3	1.3	0.3	0.0	0.2	0.0	9.7
	2	1.1	0.2	1.2	0.1	85.8	1.1	0.3	0.0	0.0	0.0	10.2
	3	1.2	0.1	1.6	0.0	85.1	1.0	0.3	0.0	0.0	0.0	10.7
+ ADH-20	1	0.6	0.8	0.4	0.0	0.1	0.0	86.0	1.5	0.3	0.3	10.1
	2	0.9	2.2	2.2	0.0	0.0	0.0	81.4	1.3	0.1	0.2	11.7
	3	0.1	0.2	0.3	0.0	0.1	0.0	87.2	1.1	0.6	0.3	10.1

Conditions: **UPO-step:** *Mor*UPO (3.75 mg/mL), H_2O_2 (0.1 eq./h for 22 h), KP_i-buffer (300 mM), pH 7, 30 °C, 600 rpm, 24 h, final volume 990 µL. **ER-step:** addition of ER (3 mg), GDH-101 (1.5 mg), NAD^+ (2 mol%), D-Glc (1.1 eq.), 30 °C, 900 rpm, 20 h, final volume 1245 µL. **ADH-step:** addition of ADH (3 mg), GDH-101 (1.5 mg), NAD^+ (2 mol%), D-Glc (1.1 eq.), 30 °C, 900 rpm, 24 h, final volume 1500 µL.

GC chromatograms

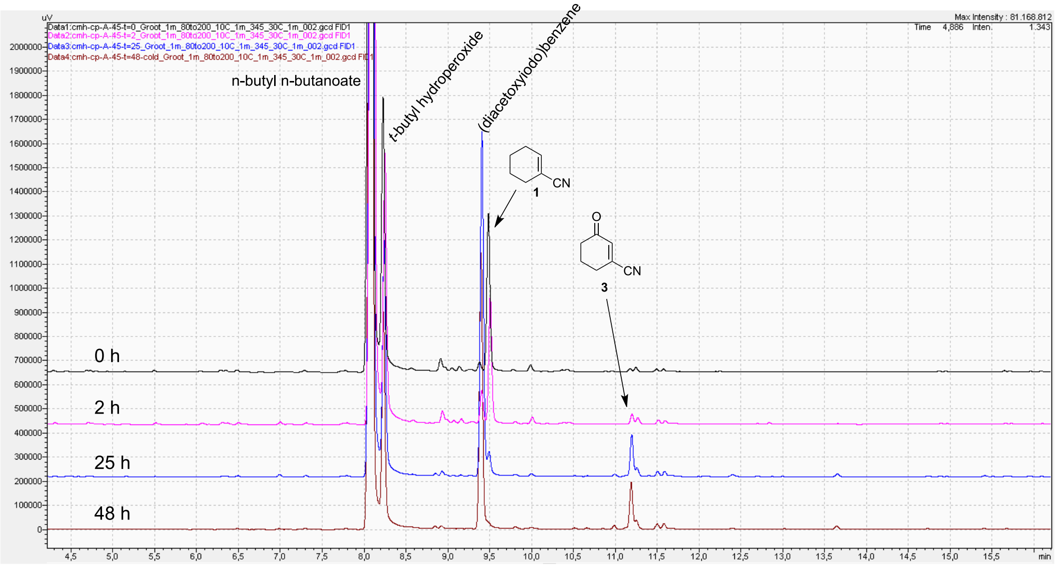

Figure S6. Stacked GC chromatograms of the time course of the allylic oxidation of **1** using (diacetoxyiodo)benzene and *t*-butyl hydroperoxide (CP-Sil 8 CB).

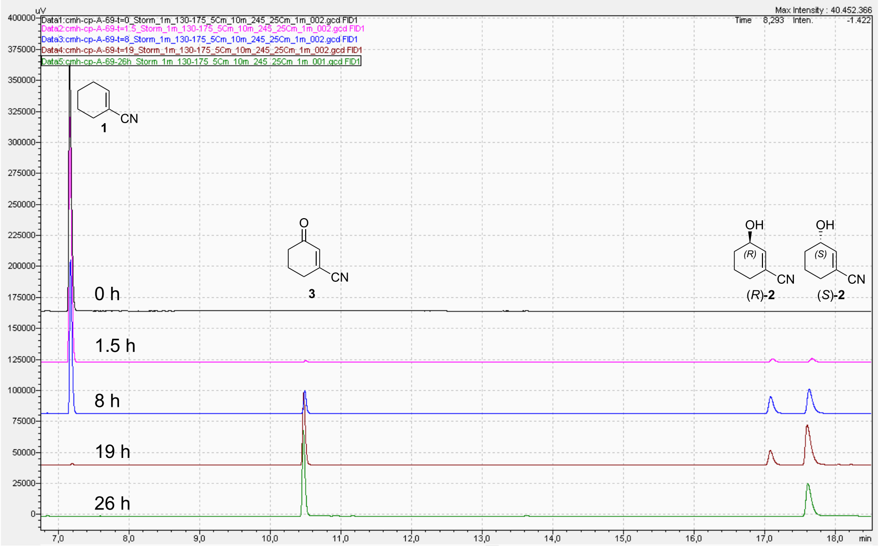

Figure S7. Stacked GC chromatograms of the time course of the allylic oxidation of **1** using rAaeUPO and H_2O_2 (Hydrodex β-6TBDM).

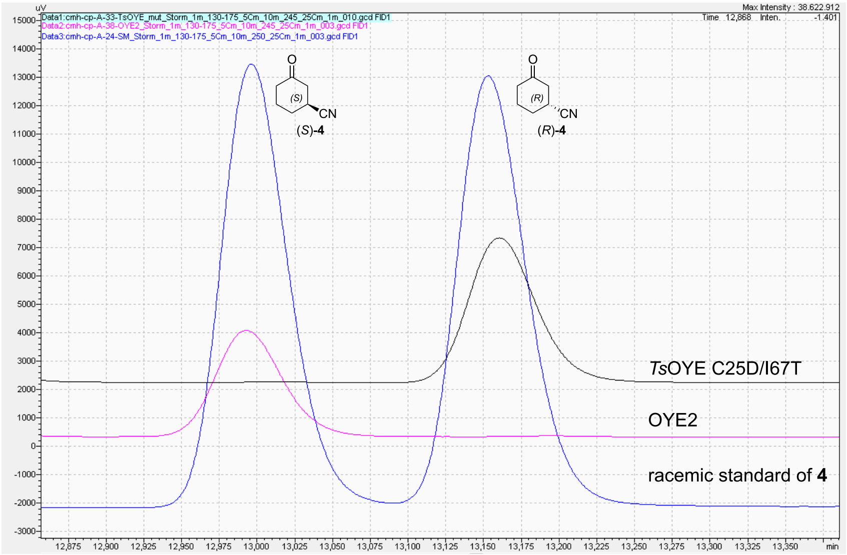

Figure S8. GC chromatograms for the assignment of stereoisomers for intermediate **4** (Hydrodex β-6TBDM)

Assignment of stereoisomers for final product 5 (Hydrodex β-6TBDM)

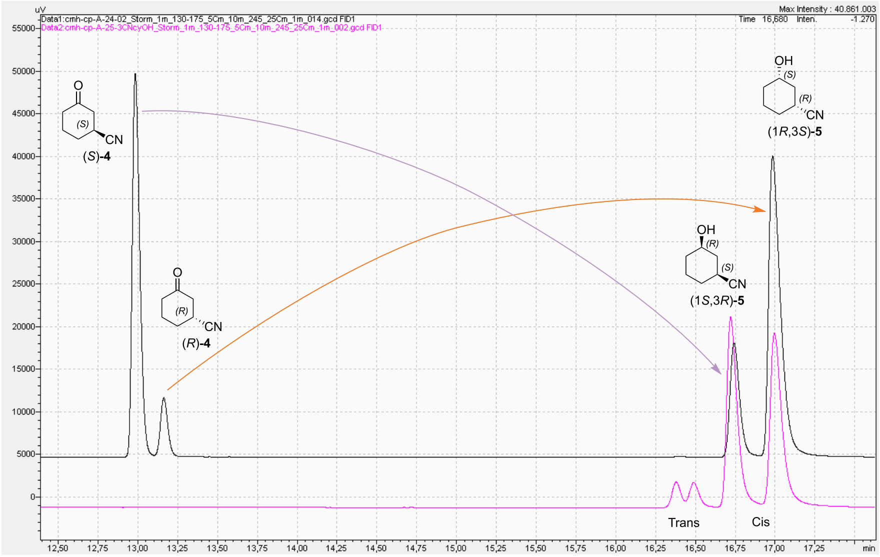

Figure S9. GC chromatograms of product **5** obtained using $NaBH_4$ (pink), and biotransformation of racemic **4** using GDH-101/$NADP^+$ (black).

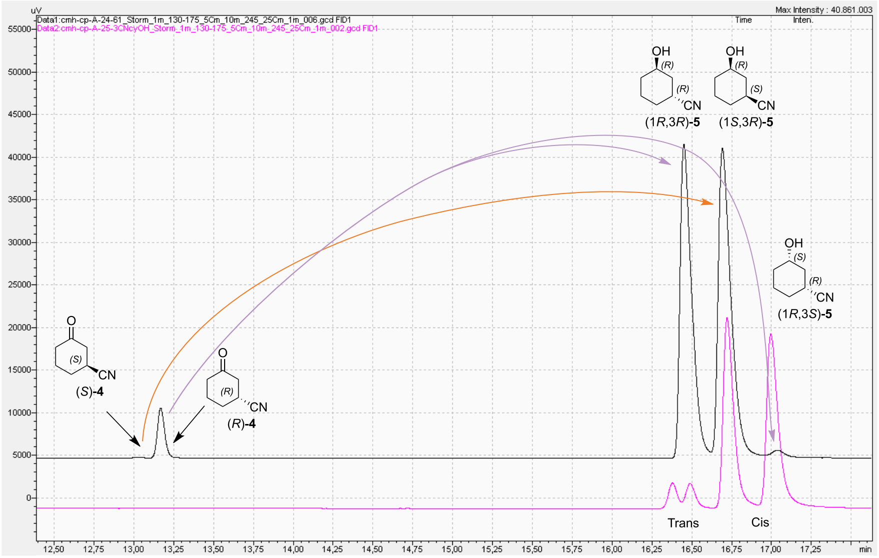

Figure S10. GC chromatograms of product **5** obtained using $NaBH_4$ (pink), and biotransformation of racemic **4** using ADH-61/NAD^+ (black)

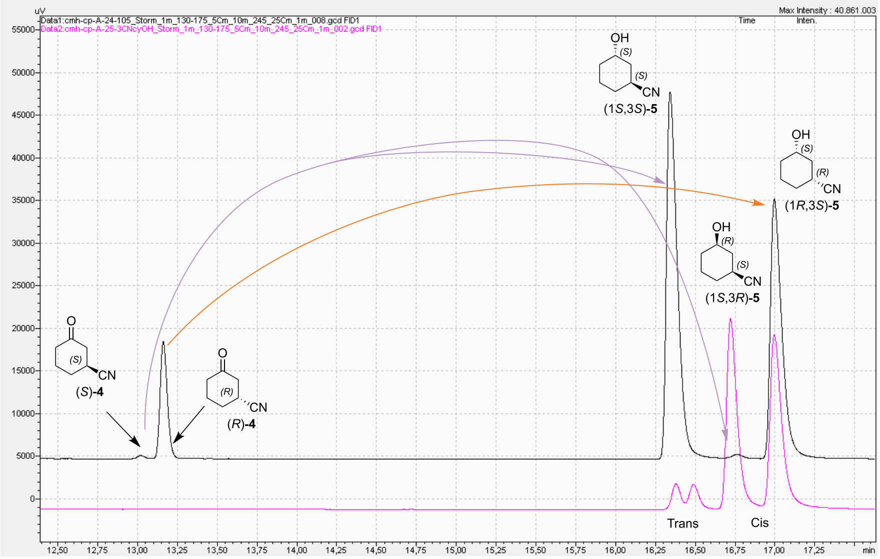

Figure S11. GC chromatograms of product **5** obtained using $NaBH_4$ (pink), and biotransformation of racemic **4** using ADH-105/NAD^+ (black)

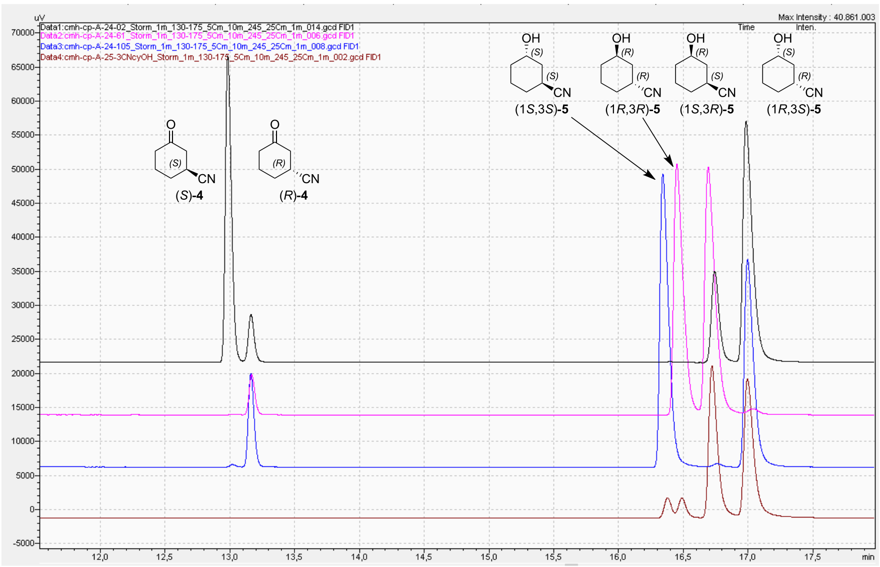

Figure S12. GC chromatograms of product **5** obtained using $NaBH_4$ (brown), and biotransformation of racemic **4** using ADH-105 (blue), ADH-61 (pink), and GDH-101 (black).

Representative chromatograms of the complete cascade from 1 to 5 (Hydrodex β-6TBDM, adjusted method)

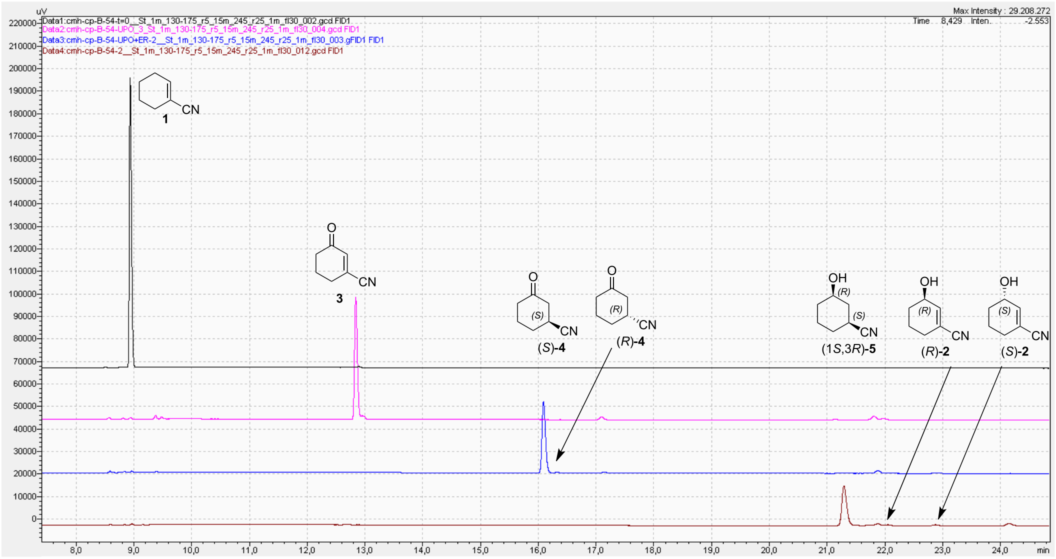

Figure S13. GC chromatograms of cascade stopped at each stage, t=0 (black), after UPO (pink), after ER (blue), and after ADH (brown).

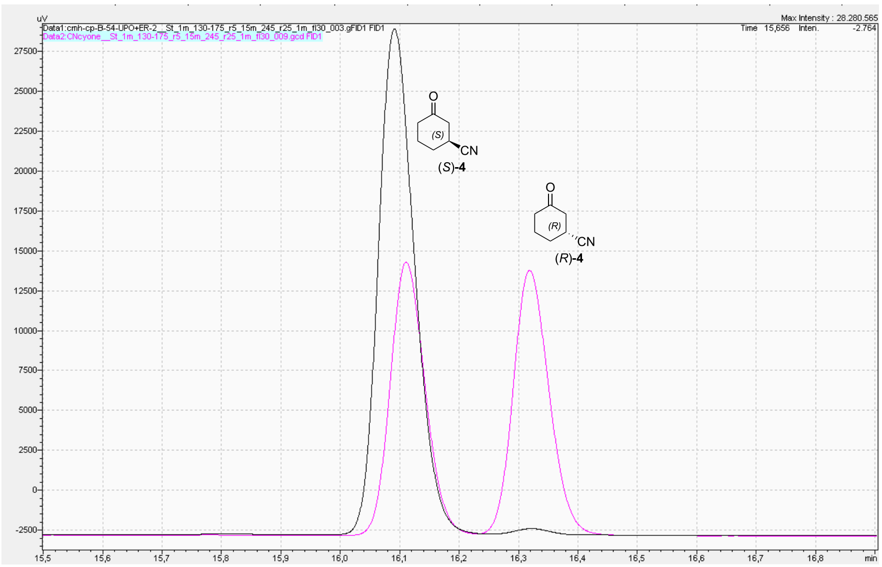

Figure S14. GC chromatograms of cascade stopped after ER step (black) and authentic standard of racemic **4** (pink).

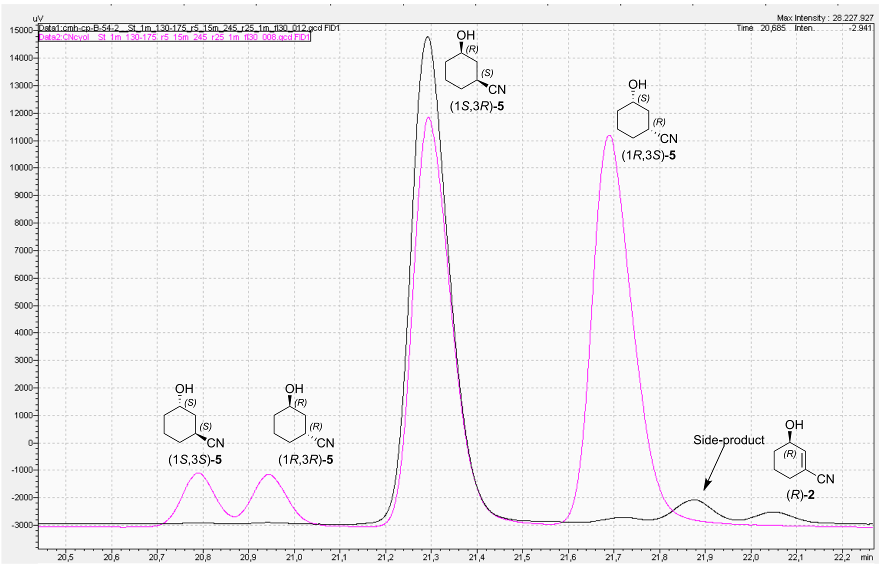

Figure S15. GC chromatograms of cascade stopped after ADH step (black) and **5** produced with $NaBH_4$.

NMR spectra

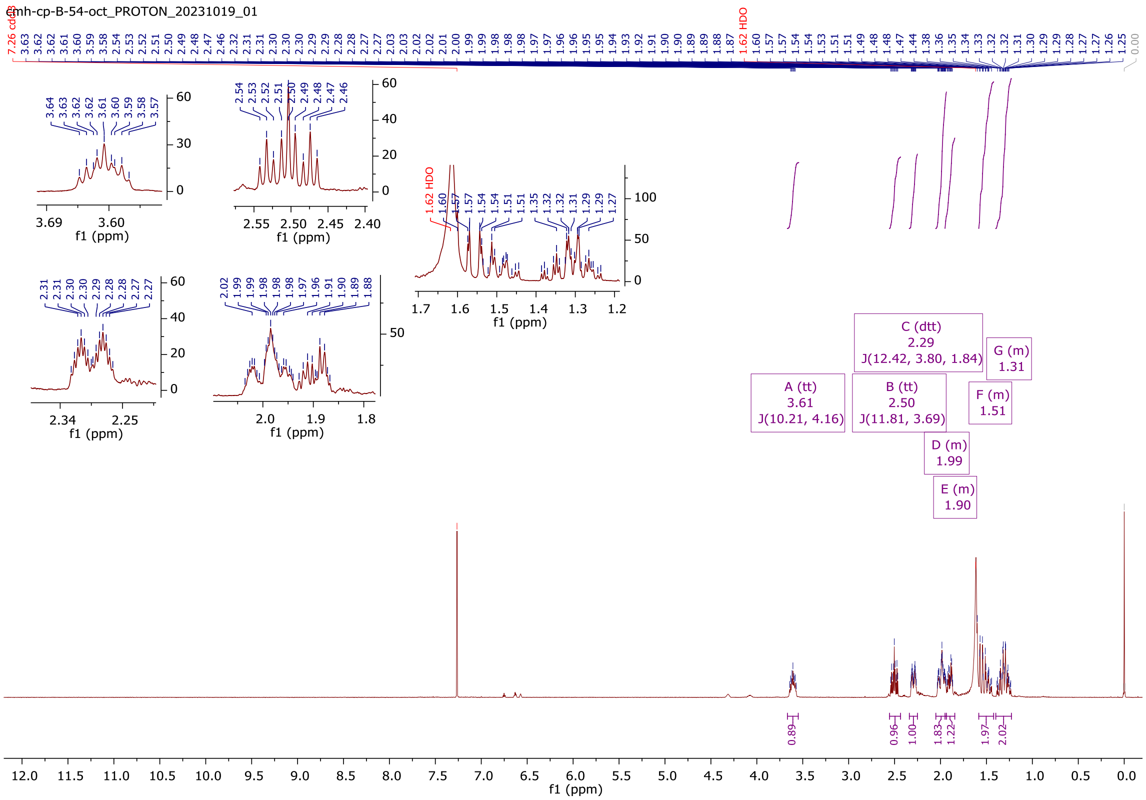

Figure S16. ^{1}H-NMR spectrum of the cascade after extraction into $CDCl_3$.

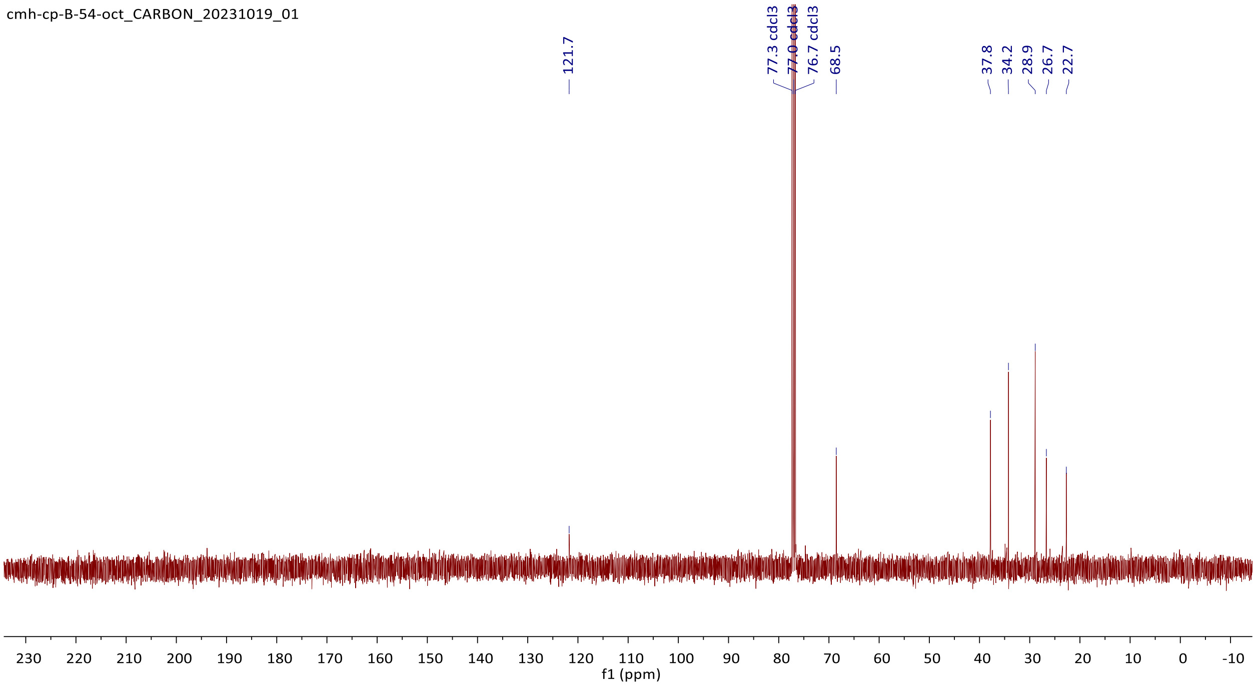

Figure S17. ^{13}C-NMR spectrum of the cascade after extraction into $CDCl_3$.

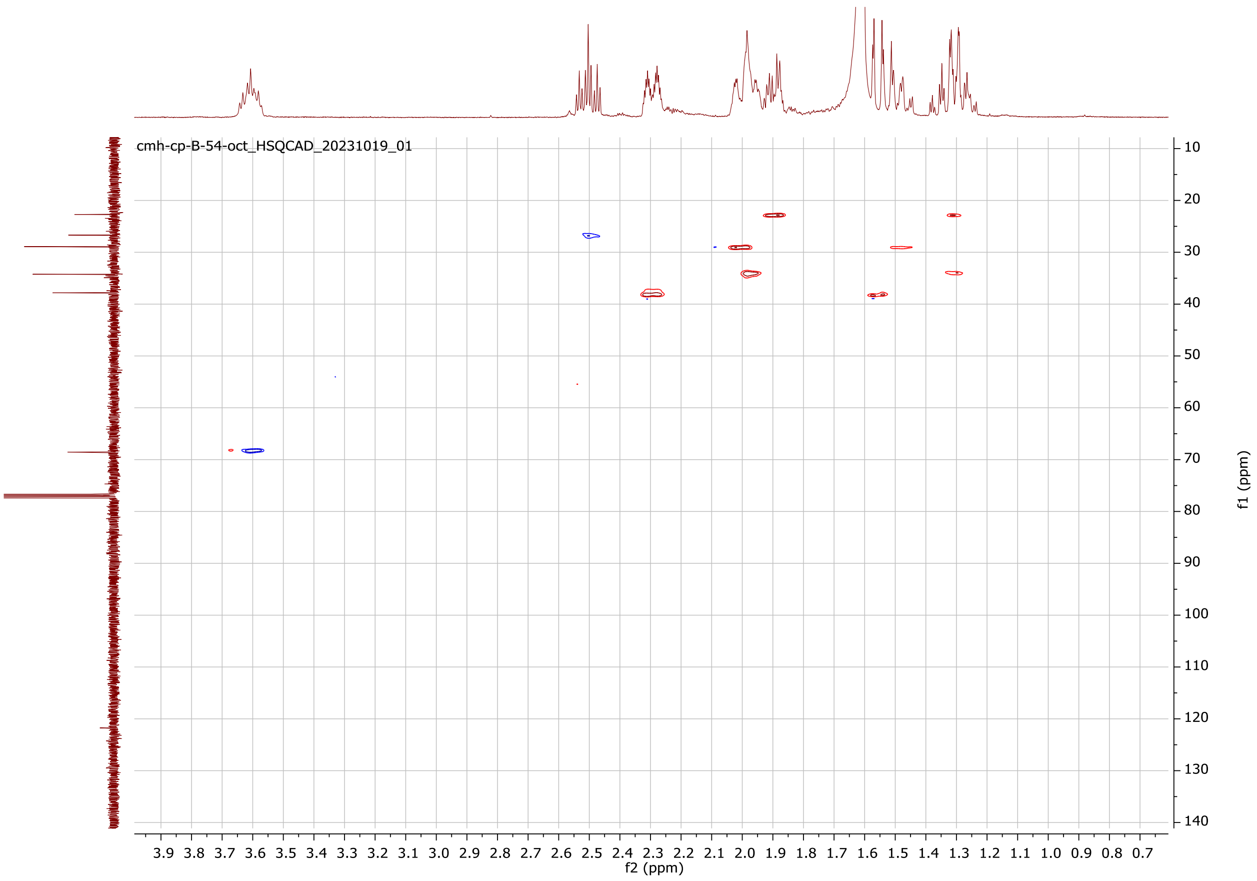

Figure S18. Multiplicity edited ^{1}H-^{13}C-HSQC spectrum of the cascade after extraction into $CDCl_3$.

References

(1) Ebner, K.; Pfeifenberger, L. J.; Rinnofner, C.; Schusterbauer, V.; Glieder, A.; Winkler, M. Discovery and heterologous expression of unspecific peroxygenases. *Catalysts* **2023**, *13*, 206.

(2) Tonin, F.; Tieves, F.; Willot, S.; van Troost, A.; van Oosten, R.; Breestraat, S.; van Pelt, S.; Alcalde, M.; Hollmann, F. Pilot-scale production of peroxygenase from *Agrocybe aegerita*. *Org. Process Res. Dev.* **2021**, *25*, 1414-1418.

(3) Jongkind, E. P. J.; Fossey-Jouenne, A.; Mayol, O.; Zaparucha, A.; Vergne-Vaxelaire, C.; Paul, C. E. Synthesis of chiral amines via a bi-enzymatic cascade using an ene-reductase and amine dehydrogenase. *ChemCatChem* **2021**, *14*, e202101576.

(4) Ribeaucourt, D.; Höfler, G. T.; Yemloul, M.; Bissaro, B.; Lambert, F.; Berrin, J.-G.; Lafond, M.; Paul, C. E. Tunable production of (*R*)- or (*S*)-citronellal from geraniol via a bienzymatic cascade using a copper radical alcohol oxidase and Old Yellow Enzyme. *ACS Catal.* **2022**, *12*, 1111-1116.

(5) Zhao, Y.; Yeung, Y. Y. An unprecedented method for the generation of *tert*-butylperoxy radical using DIB/TBHP protocol: Solvent effect and application on allylic oxidation. *Org. Lett.* **2010**, *12*, 2128-2131.

(6) Catino, A. J.; Forslund, R. E.; Doyle, M. P. Dirhodium(II) caprolactamate: an exceptional catalyst for allylic oxidation. *J. Am. Chem. Soc.* **2004**, *126*, 13622-13623.

(7) Shing, T. K. M.; Yeung, Y. Y.; Su, P. L. Mild manganese(III) acetate catalyzed allylic oxidation: Application to simple and complex alkenes. *Organic Letters* **2006**, *8*, 3149-3151.

(8) Zhang, W.; Gacs, J.; Arends, I. W. C. E.; Hollmann, F. Selective photooxidation reactions using water-soluble anthraquinone photocatalysts. *ChemCatChem* **2017**, *9*, 3821-3826.

(9) Lechner, R.; Kummel, S.; Konig, B. Visible light flavin photo-oxidation of methylbenzenes, styrenes and phenylacetic acids. *Photochem. Photobiol. Sci.* **2010**, *9*, 1367-1377.

(10) Srivastava, V.; Singh, P. K.; Singh, P. P. Visible light promoted allylic C-H oxidation. *Croat. Chem. Acta* **2017**, *90*, 435-441.
